# Genome-wide association study reveals new insights into the heritability and genetic correlates of developmental dyslexia

**DOI:** 10.1038/s41380-020-00898-x

**Published:** 2020-10-14

**Authors:** Alessandro Gialluisi, Till F. M. Andlauer, Nazanin Mirza-Schreiber, Kristina Moll, Jessica Becker, Per Hoffmann, Kerstin U. Ludwig, Darina Czamara, Beate St Pourcain, Ferenc Honbolygó, Dénes Tóth, Valéria Csépe, Guillaume Huguet, Yves Chaix, Stephanie Iannuzzi, Jean-Francois Demonet, Andrew P. Morris, Jacqueline Hulslander, Erik G. Willcutt, John C. DeFries, Richard K. Olson, Shelley D. Smith, Bruce F. Pennington, Anniek Vaessen, Urs Maurer, Heikki Lyytinen, Myriam Peyrard-Janvid, Paavo H. T. Leppänen, Daniel Brandeis, Milene Bonte, John F. Stein, Joel B. Talcott, Fabien Fauchereau, Arndt Wilcke, Holger Kirsten, Bent Müller, Clyde Francks, Thomas Bourgeron, Anthony P. Monaco, Franck Ramus, Karin Landerl, Juha Kere, Thomas S. Scerri, Silvia Paracchini, Simon E. Fisher, Johannes Schumacher, Markus M. Nöthen, Bertram Müller-Myhsok, Gerd Schulte-Körne

**Affiliations:** 1 Department of Translational Research in Psychiatry, Max Planck Institute of Psychiatry, Munich, Germany; 2 Munich Cluster for Systems Neurology (SyNergy), Munich, Germany; 3 Department of Epidemiology and Prevention, IRCCS Istituto Neurologico Mediterraneo Neuromed, Pozzilli, Italy; 4 Department of Neurology, Klinikum rechts der Isar, School of Medicine, Technical University of Munich, Munich, Germany; 5 Institute of Neurogenomics, Helmholtz Zentrum München, Neuherberg, Germany; 6 Department of Child and Adolescent Psychiatry, Psychosomatic, and Psychotherapy, Ludwig-Maximilians University, Munich, Germany; 7 Department of Genomics, Life and Brain Center, Institute of Human Genetics, University of Bonn, Bonn, Germany; 8 Language and Genetics Department, Max Planck Institute for Psycholinguistics and Donders Institute for Brain, Cognition and Behaviour, Radboud University, Nijmegen, The Netherlands; 9 MRC Integrative Epidemiology Unit, University of Bristol, Bristol, UK; 10 Brain Imaging Centre, Research Centre of Natural Sciences of the Hungarian Academy of Sciences, Budapest, Hungary; 11 Human Genetics and Cognitive Functions Unit, Institut Pasteur and University Paris Diderot, Sorbonne Paris Cité, Paris, France; 12 ToNIC, Toulouse NeuroImaging Center, Université de Toulouse, Inserm, UPS, Toulouse, France; 13 Children’s Hospital, Purpan University Hospital, Toulouse, France; 14 Leenaards Memory Centre, Department of Clinical Neurosciences Lausanne University Hospital (CHUV), University of Lausanne, Lausanne, Switzerland; 15 Department of Biostatistics, University of Liverpool, Liverpool, UK; 16 Centre for Genetics and Genomics Versus Arthritis, Centre for Musculoskeletal Research, University of Manchester, Manchester, UK; 17 Wellcome Centre for Human Genetics, University of Oxford, Oxford, UK; 18 Institute for Behavioral Genetics and Department of Psychology and Neuroscience, University of Colorado Boulder, Boulder, CO USA; 19 Department of Neurological Sciences, University of Nebraska Medical Center, Omaha, NE USA; 20 Developmental Neuropsychology Lab and Clinic, Department of Psychology, University of Denver, Denver, CO USA; 21 Department of Cognitive Neuroscience, Faculty of Psychology and Neuroscience and Maastricht Brain Imaging Center (M-BIC), Maastricht University, Maastricht, The Netherlands; 22 Department of Psychology, The Chinese University of Hong Kong, Shatin, N.T., Hong Kong; 23 Centre for Research on Learning and Teaching, Department of Psychology, University of Jyväskylä, Jyväskylä, Finland; 24 Department of Biosciences and Nutrition, Karolinska Institutet, Huddinge, Sweden; 25 Department of Child and Adolescent Psychiatry and Psychotherapy, Psychiatric Hospital, University of Zurich, Zurich, Switzerland; 26 Zurich Center for Integrative Human Physiology (ZIHP), University of Zurich and ETH Zurich, Zurich, Switzerland; 27 Neuroscience Center Zurich, University of Zurich and ETH Zurich, Zurich, Switzerland; 28 Department of Child and Adolescent Psychiatry and Psychotherapy, Central Institute of Mental Health, Medical Faculty Mannheim, Heidelberg University, Mannheim, Germany; 29 Department of Physiology, University of Oxford, Oxford, UK; 30 School of Life and Health Sciences, Aston University, Birmingham, UK; 31 Cognitive Genetics Unit, Fraunhofer Institute for Cell Therapy and Immunology, Leipzig, Germany; 32 Institute for Medical Informatics, Statistics and Epidemiology and LIFE—Leipzig Research Center for Civilization Diseases, University of Leipzig, Leipzig, Germany; 33 Tufts University, Medford, MA USA; 34 Laboratoire de Sciences Cognitives et Psycholinguistique, Ecole Normale Supérieure, CNRS, EHESS, PSL University, Paris, France; 35 Institute of Psychology, University of Graz and BioTechMed, Graz, Austria; 36 Stem Cells and Metabolism Research Program, Biomedicum, Folkhälsan Institute of Genetics, University of Helsinki, Helsinki, Finland; 37 The Walter and Eliza Hall Institute of Medical Research, Melbourne University, Melbourne, VIC Australia; 38 School of Medicine, University of St Andrews, St Andrews, UK; 39 Institute of Translational Medicine, University of Liverpool, Liverpool, UK

**Keywords:** Genetics, Neuroscience, Psychiatric disorders

## Abstract

Developmental dyslexia (DD) is a learning disorder affecting the ability to read, with a heritability of 40–60%. A notable part of this heritability remains unexplained, and large genetic studies are warranted to identify new susceptibility genes and clarify the genetic bases of dyslexia. We carried out a genome-wide association study (GWAS) on 2274 dyslexia cases and 6272 controls, testing associations at the single variant, gene, and pathway level, and estimating heritability using single-nucleotide polymorphism (SNP) data. We also calculated polygenic scores (PGSs) based on large-scale GWAS data for different neuropsychiatric disorders and cortical brain measures, educational attainment, and fluid intelligence, testing them for association with dyslexia status in our sample. We observed statistically significant (*p*  < 2.8 × 10^−6^) enrichment of associations at the gene level, for *LOC388780* (20p13; uncharacterized gene), and for *VEPH1* (3q25), a gene implicated in brain development. We estimated an SNP-based heritability of 20–25% for DD, and observed significant associations of dyslexia risk with PGSs for attention deficit hyperactivity disorder (at *p*_*T*_ = 0.05 in the training GWAS: OR = 1.23[1.16; 1.30] per standard deviation increase; *p*  = 8 × 10^−13^), bipolar disorder (1.53[1.44; 1.63]; *p* = 1 × 10^−43^), schizophrenia (1.36[1.28; 1.45]; *p* = 4 × 10^−22^), psychiatric cross-disorder susceptibility (1.23[1.16; 1.30]; *p* = 3 × 10^−12^), cortical thickness of the transverse temporal gyrus (0.90[0.86; 0.96]; *p* = 5 × 10^−4^), educational attainment (0.86[0.82; 0.91]; *p* = 2 × 10^−7^), and intelligence (0.72[0.68; 0.76]; *p* = 9 × 10^−29^). This study suggests an important contribution of common genetic variants to dyslexia risk, and novel genomic overlaps with psychiatric conditions like bipolar disorder, schizophrenia, and cross-disorder susceptibility. Moreover, it revealed the presence of shared genetic foundations with a neural correlate previously implicated in dyslexia by neuroimaging evidence.

## Introduction

Developmental dyslexia (DD) is a specific learning disorder affecting the ability to read that is not better accounted for by intellectual disabilities, uncorrected visual or auditory acuity, other mental or neurological disorders, or inadequate educational instruction [1]. People with dyslexia show difficulties in accurate and/or fluent word recognition, decoding, spelling, and/or reading comprehension [2]. The prevalence of DD is reported to be around 5–10% among school-aged children, depending on the criteria used for diagnosis [3]. DD tends to recur in families [4, 5] and most twin studies have reported a heritability (*h*^2^) between 40 and 60% [2, 6]. A similar range of heritability has been reported for several cognitive skills representing/underlying reading ability, such as word reading, spelling, and phoneme awareness (*h*^2^ ~40–70%) [7–9]. Of note, a large proportion of this heritability remains unexplained, and DD shows a complex architecture, with multiple genetic and environmental factors playing a role in its aetiology [10].

Linkage and candidate gene association studies have identified a small number of candidate susceptibility genes, most of which have been associated not only with dyslexia, but also with continuous interindividual variation in relevant cognitive skills like word reading, spelling, and others (as reviewed in [11–13]). The most robust candidate genes identified so far include *DYX1C1* (15q21) [14], *DCDC2* and *KIAA0319* (6p22.3) [15–18], *GCFC2* and *MRPL19* (2p12) [19], and *ROBO1* (3p12.3-p12.3) [20–22]. DD and reading-related cognitive traits have also been investigated via genome-wide association studies (GWAS), which involve analyses of many single-nucleotide polymorphisms (SNPs) spread across the genome. A few such studies have been reported, using either a case-control design [23–25] or a continuous trait analysis approach [26–30]. However, only two of these studies identified associations that met criteria for genome-wide significance [27, 28]. The first was a GWAS of multiple cognitive skills related to reading ability, which revealed a genome-wide significant association at rs17663182 (*MIR924HG*; 18q12.2) with rapid automatized naming (RAN), in nine cohorts of reading-impaired and typically developing subjects of European ancestry (maximum *N* = 3468) [28]. More recently, in a north-American cohort of non-European ancestry (*N* = 1331), Truong et al. [27] identified a genome-wide significant multivariate association of rs1555839 (10q23.31; upstream from the *RPL7P34* gene) with RAN and rapid alternating stimulus, and replicated the association with RAN in an independent cohort of European ancestry [27].

Here, we carried out a case-control GWAS meta-analysis involving 2274 dyslexia cases and 6272 controls from nine different countries that partly overlap with those from the prior Gialluisi et al. study of continuous traits (≤2500 overlapping samples) [28]. We performed association testing at the single variant, gene, and pathway level, and estimated SNP-based heritability. Moreover, we analyzed associations of polygenic scores (PGS) derived from large-scale GWAS data from other related neuropsychiatric disorders, as well as intelligence, educational attainment, and cortical brain measures.

## Subjects and methods

### Datasets

The datasets involved in the present study were collected in nine different populations of European ancestry, with six different languages (see Table 1). Subsets have already been tested for association with continuous reading-related traits [28]. Ethical approval was obtained for each cohort at the local level, and written informed consent was obtained for all the participants or their parents.

**Table 1:** General descriptive statistics and recruitment criteria of the datasets analyzed in the study.

Dataset	Recruitment	Language	Age range, years (mean, SD)^b^	IQ inclusion criteria	*N* after QC (cases:controls)
AGS^a^	Unrelated cases and controls	German	8–19 (10.7, 2.4)	Age-appropriate WISC Block Design score [99, 100] ≥7 Age-appropriate WISC Similarities score [99, 100] ≥6	1454 (1047:407)
Finland		Finnish			324 (161:163)
France		French			163 (106:57)
Hungary		Hungarian			241 (90:151)
Netherlands		Dutch			284 (136:148)
ENall1^b^	Related cases from Colorado + related cases from UK (Oxford) + unrelated unscreened controls from UK general population (WTCC2_1958)	English	8–19 (11.5, 2.7)	Colorado: average score of age-adjusted WISC-R/WAIS-R verbal IQ and performance IQ, multiple subtests) [101] ≥80 [28] UK: average of age-adjusted standardized BAS/WAIS-R similarities subtest and BAS matrices subtest score [102, 103] ≥80 [28]	3313 (554:2759)
ENall2^b^	Unrelated cases from UK (Cardiff) + unrelated unscreened controls from UK general population (WTCC2_NBS)	English	5–31 (11.8, 3.6)	Average of age-adjusted standardized BAS/WAIS-R similarities subtest and BAS matrices subtest score [102, 103] ≥80 [28]	2767 (180:2587)

*IQ* intelligence quotient, *DD* developmental dyslexia, *WISC* Wechsler Intelligence Scale for Children, *WAIS* Wechsler Adult Intelligence Scale—Revised, *BAS* British Ability Scale.

^a^Austria–Germany–Switzerland.

^b^In the English-speaking datasets (i.e., ENall1 and ENall2) age and IQ information refers only to cases, since no such data were available from the general population control cohorts.

Unrelated DD cases and controls with IQ in the normal range were recruited in Austria (*N* = 374), Finland (*N* = 336), France (*N* = 165), Germany (*N* = 1454), Hungary (*N* = 243), The Netherlands (*N* = 311), and Switzerland (*N* = 67) (see Table 1). DD cases were defined as participants showing low performance on tests of word reading (standardized score ≤−1.25), with the exception of 148 German cases, which were defined based on a ≥1.5 standard deviation discrepancy between the observed and expected spelling score based on their IQ (see [31, 32] and Supplementary methods). Controls were defined as individuals with standardized word reading scores >−0.85 [33, 34].

Samples from Austria, Germany, and Switzerland were merged together into a single dataset (hereafter called AGS), since they shared language and genetic ancestry [28]. Two additional datasets were included in the study, made up of native English speakers. One of them consisted of DD cases selected from two sibling-based cohorts, namely the Colorado Reading Disability Cohort [26, 35] and an independent cohort from Oxford, UK [28, 36]. These cases were merged to form a single case-control dataset with unscreened controls from the Wellcome Trust Case Control Consortium 2 (WTCCC2) 1958 British birth cohort (WTCCC2_1958), a sample of sequential live births in the UK during 1 week in 1958 [37]. The other English-speaking dataset consisted of unrelated DD cases recruited in Cardiff, UK and the WTCCC2 National Blood Service (WTCCC2_NBS) cohort, a collection of subjects who have donated blood to the UK blood service. These datasets, hereafter called ENall1 (*N* = 3531) and ENall2 (*N* = 2947), met the same word reading-based inclusion criteria as above for cases, while controls were unscreened, as in other prominent studies [38, 39].

### Genotype quality control (QC) and imputation

Genotyping array platforms used for the different datasets are reported in Table S1a. These included Illumina HumanHap 300k, 550k, 660k, OmniExpress Human CoreExome and BeadChips, and Illumina 1.2 M chips. Genotype QC was carried out, as previously described [28], in PLINK v1.90b3s [40] and QCTOOL v1.4 (see URLs). Briefly, SNPs were excluded if they showed a variant call rate <98%, a minor allele frequency (MAF) <5%, or a Hardy–Weinberg equilibrium (HWE) exact test *p* value < 10^−6^. Samples showing a genotyping rate <98%, mismatches between genetic and pedigree-based sex, cryptic relatedness (in datasets of unrelated subjects), or identity-by-descent not corresponding to the available pedigree information (in datasets including related cases) were also discarded. Similarly, we discarded genetic ancestry outliers detected in a multidimensional scaling (MDS) analysis of pairwise genetic distance and samples with extreme genome-wide heterozygosity values (see Table S1b).

For imputation, genotypes of autosomal SNPs were aligned to the 1000 Genomes phase I v3 reference panel (ALL populations, June 2014 release) [41] and pre-phased using SHAPEIT v2 (r837) [42]. Imputation was then performed using IMPUTE2 v2.3.2 [43] in 5 Mb chunks with 500 kb buffers, filtering out variants that were monomorphic in the 1000 Genomes EUR (European) samples. Chunks with <51 genotyped variants or concordance rates <92% were fused with neighbouring chunks and re-imputed. Finally, imputed variants (genotype probabilities) were filtered for IMPUTE2 INFO metric ≥0.8, as well as MAF and HWE thresholds as above. We re-evaluated genetic ancestry and genome-wide heterozygosity outliers after imputation and observed substantial concordance with pre-imputation QC. After QC, 2274 dyslexia cases and 6272 controls were left for analysis (see Tables S1c, d for a power computation).

### Genetic association test and meta-analysis

After genotype QC and imputation, we tested autosomal variant allelic dosages for association with case-control status within each dataset. In all the datasets except ENall1, we ran association tests through logistic regression in PLINK, using the first ten genetic ancestry (MDS) components as covariates. To account for the genetic relationship among related subjects in ENall1, we modelled a generalized linear mixed-effects model association test through FastLMM v2.07 [44], using a genetic relationship matrix as a random effect, while disabling normalization to unit variance for tested SNPs. Then we combined the results of the association tests in the different datasets through a fixed-effects sample size-based meta-analysis in METAL v25-03-2011 (“Stouffer” method) [45]. This was done in order to overcome the heterogeneity of scales of the association tests used in the different datasets. The genome-wide significance threshold was set to *α* = 5 × 10^−8^. To obtain an estimate of the odds ratio (OR) for the top association identified, we performed a Wald association test in the ENall1 dataset through a logistic mixed model approach in GMMAT [46], which was not possible to perform at the genome-wide level due to the high computational load implied. We then meta-analyzed the resulting association statistics across datasets, through a fixed-effects inverse variance-weighted method in METAL [45].

### Gene- and pathway-based enrichment tests

We performed a gene-based association analysis on the results of the GWAS meta-analysis in MAGMA v1.06 [47]. First, we assigned genetic variants to protein-coding genes based on their position according to the NCBI 37.3 (*hg19*) build, extending the region of annotation to 10 kb from the 3′-/5′-UTR (untranslated region). In total, 18,013 genes (out of 19,427 genes available) included at least one variant that passed internal QC and were thus tested for enrichment of single-variant associations, using default settings. For this analysis, we set a genome-wide significance threshold *α* = 2.8 × 10^−6^, correcting for 18,013 genes tested.

We used the results of the gene-based association analysis to carry out a pathway-based enrichment test for associations with DD, through a competitive gene-set analysis in MAGMA v1.06. We tested for enrichment 1329 canonical pathways (i.e., classical representations of biological processes compiled by domain experts) from the Molecular Signatures Database website (MSigDB v5.2, collection C2, subcollection CP; see URLs). To correct enrichment statistics for testing of multiple pathways, we used an adaptive permutation procedure with default settings (up to a maximum of 10,000 permutations). Hence, in this analysis we set the significance threshold to *α* = 0.05.

### Estimation of heritability

We used the summary statistics from the DD case-control GWAS to compute SNP-based heritability of the disorder, through LD score regression [48, 49]. For this analysis, we used only common SNPs tested in the GWAS and present in the HapMap 3 reference panel [50], excluding the MHC region, since these variants show a good imputation quality (*r*^2^ > 0.9) in most studies. All the analyses presented below were performed on these variants (1,025,494 SNPs), using LD information based on the 1000 G phase 1 v3 EUR panel (see URLs).

We first computed the proportion of genetic variance explained by all SNPs mentioned above on the observed scale, and then repeated the analysis using a liability threshold model, i.e., assuming that the binary trait that we use is determined by an unobserved normally distributed liability threshold [48, 49]. This analysis requires specification of the proportion of cases in the GWAS (27%), and the estimated prevalence of the disorder in the reference population, which has been reported to be 5–10% among school-aged children [3]. Hence, we carried out the analysis using the limits of this prevalence range, namely 0.05 and 0.10, respectively.

To extrapolate biological information from our GWAS summary statistics, we computed partitioned heritability for 53 overlapping functional annotation categories identified in the genome [51], irrespective of the cell types analyzed (baseline model). These annotations include DNase I hypersensitivity sites, coding regions, untranslated regions, enhancers, promoters and several histone marks as defined by different public resources (see “Results” section and [51] for a complete list). Similarly, we carried out a stratified LD score regression using only central nervous system (CNS) cell-specific annotations of four histone marks—H3K4me1, H3K4me3, H3K9ac, and H3K27ac—to identify a specific enrichment of functional elements associated with transcriptional activity in these cells. We performed this analysis both for all the CNS cells pooled together and singularly for each cell type available in brain tissues, while correcting for the contribution of all functional annotation categories previously tested in the baseline model, as suggested by the developer [51]. Thereby, we could identify the contribution of common variants annotated to histone marks which are specifically enriched in nervous cells. Finally, we computed partitioned heritability for diverse sets of genes whose expression is specifically enriched in 13 different brain regions, based on RNA-seq data from the Genotype-Tissue Expression portal (GTEx v6) [52, 53]. The brain regions available included amygdala, anterior cingulate cortex, caudate nucleus, cerebellar hemispheres, cerebellum, cortex, frontal cortex, hippocampus, hypothalamus, nucleus accumbens, putamen, spinal cord, and substantia nigra.

### Polygenic score (PGS) analyses

#### Genetic liability to neuropsychiatric disorders, intelligence and education

We investigated potential genetic links between dyslexia and related and/or comorbid neuropsychiatric disorders, including attention deficit hyperactivity disorder (ADHD) [54–56], autism spectrum disorder (ASD) [57], major depressive disorder (MDD) [58], bipolar disorder (BD) [59], and schizophrenia (SCZ) [60], as well as with genetic liability shared across different neuropsychiatric disorders, including ADHD, ASD, BD, MDD, SCZ, anorexia nervosa, obsessive-compulsive disorder and Tourette syndrome [61]. Moreover, we tested association with fluid intelligence [62] and educational attainment (years of education completed, EduYears) [63], which are phenotypically correlated with reading ability [64, 65]. To this end, we performed a PGS analysis in our sample using summary statistics available from previous independent GWAS studies of the other traits of interest (hereafter called training GWAS) [61–63, 66–70]. PGSs were computed with PRSice-2 v2.2.11 [71], using only summary statistics based on samples of European ancestry in the training GWAS and quality controlled variants in a random extraction of one individual per family from our dyslexia (target) GWAS (MAF ≥ 5%; HWE *p* ≥ 10^−6^; variant call rate ≥95%; *N* = 8456). We further pruned SNPs through LD-clumping (pairwise *r*^2^ < 0.05 within sliding 300 kb windows) and removed those variants with discordant coordinates/alleles between the training and the target GWAS. We then computed average (default) PGS using only variants with association *p* value < 0.05 in the training GWAS (as in [28, 72, 73]), since this represents a reasonable trade-off between goodness-of-fit of the PGS and the risk of introducing noise in the model by including genetic variants meeting more lenient association thresholds. We then built generalized linear models (glm) of dyslexia vs PGS adjusted for sex and genetic ancestry (10 MDS components) in the same set of unrelated subjects used above (2184 cases and 6272 controls). To check for robustness of our findings, we repeated the analysis at different association significance thresholds in each training GWAS (with *p* < 5 × 10^−8^, 1 × 10^−5^, 0.001, 0.05, 0.1, 0.2, 0.3, 0.4, 0.5, 0.6, 0.7, 0.8, 0.9 and 1.0). A Bonferroni-corrected significance threshold was set to *α* = 4.5 × 10^−4^ for this analysis, conservatively correcting for eight (six binary neuropsychiatric and two continuous) training traits, and 14 significance thresholds tested.

#### Polygenic scores of brain cortical measures

We carried out an exploratory analysis to test associations with PGSs influencing the surface area (SA) and thickness (T) of 34 brain cortical regions (June 2020 release), recently analyzed in a GWAS involving 33,992 participants of European ancestry [74]. We tested PGSs (at *p* < 0.05 in the training GWAS) of both SA and T of all the cortical regions adjusted for global measures (total SA and average T, respectively), both separately and jointly in a multivariable setting. This choice was motivated by the fact that different structural alterations have been described in dyslexic subjects [75] and a complex brain network of different structures is thought to underlie dyslexia phenotypes and related skills [76]. To insure against potential overfitting bias in a conventional ordinary least squares regression with a high number of predictors, we applied two alternative multivariable models. First, a stepwise regression through the *stepaic()* function of the *MASS* package, which retains only variables associated with a decrease in the Akaike information criterion, representing a trade-off between goodness-of-fit and parsimony of the model. Then, an elastic net regression, using the *glmnet* and *caret* packages, as in [77]. To this end, we divided our dataset into a random training and test set (80:20 ratio), then trained the elastic net and carried out hyperparameter (*α* and *λ*) tuning in the training set, with tenfold cross-validation. Finally, we tested the performance of the optimized model, assessing classification accuracy in the independent test set (*N* = 1690). All the models involving cortical PGSs were adjusted for MDS components and sex, as explained above. For this analysis, we considered associations as statistically robust only if they showed significant and similar effect sizes across the different models tested (using a significance threshold *α* = 8.3 × 10^−4^, correcting for 60 independent cortical measures, as in [74]).

## Results

### Single-variant genome-wide associations

**Fig. 1:**
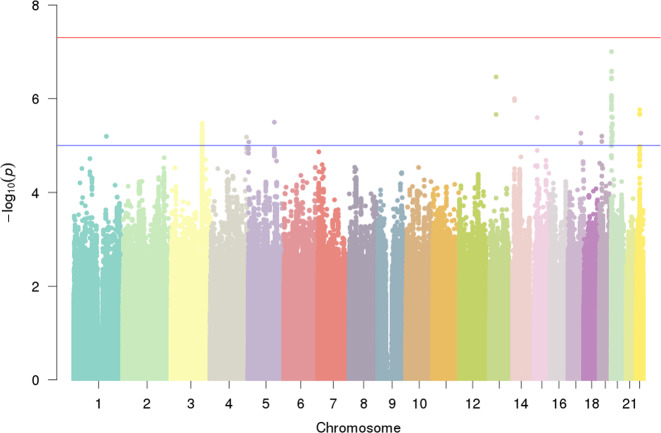
Manhattan plot of the GWAS pooled analysis. The blue and red line represent the genome-wide (*α* = 5 × 10^−8^) and suggestive significance (*α* = 1 × 10^−5^) threshold.

**Fig. 2:**
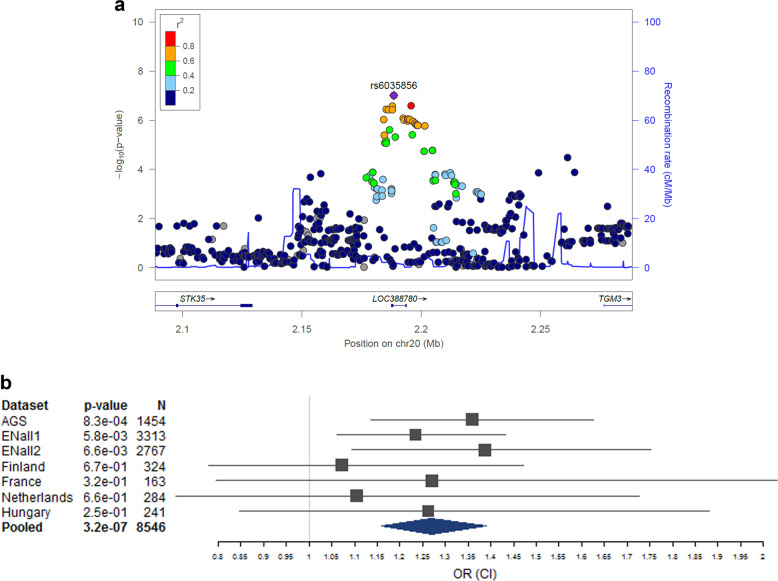
Details of the genome-wide top hit rs6035856. **a** Local association and **b** forest plot of the genome-wide top variant (rs6035856). The forest plot shows the odds ratio (OR) and 95% confidence intervals (CI) on the *x*-axis, by dataset and for the pooled analysis. Detailed OR statistics can be found in Table S2b. Note to forest plot: the sibling-based dataset ENall1 was analyzed genome-wide through linear mixed modelling (in FastLMM) for computational reasons, while its OR, as shown here, was computed via a Wald test in a logistic mixed model (GMMAT), to make it comparable to the other ORs produced through logistic regression (PLINK). Hence, the result of the pooled analysis—which here was performed through the inverse variance-based method—is slightly discrepant from the original genome-wide analysis (see Table 2).

No single-variant association with DD reached genome-wide significance (Figs. 1 and S1). The strongest single-variant associations detected in the GWAS are reported in Table 2 (*p* < 5 × 10^−7^) and, more extensively, in Table S2a (*p* < 10^−5^). The top hit was detected at rs6035856 (G/T, MAF = 0.45; *p* = 9.9 × 10^−8^), an intronic variant located within the gene *LOC388780* (chr20p13; Fig. 2a). Following logistic mixed modelling and inverse variance-based meta-analysis of the rs6035856 association, we computed an OR [confidence interval] of 1.27[1.16; 1.39] for the major allele G (*p* = 3.2 × 10^−7^). In all datasets, the major allele G was associated with increased DD risk (Fig. 2b and Table S2b). Although this SNP was not directly genotyped, it showed high quality imputation statistics across datasets (INFO metric in the range 0.89–0.95). Other SNPs in the vicinity of rs6035856 were also associated with DD (see Table 2) and were all in moderate/high LD with the top hit (*r*^*2*^ > 0.6; see Fig. 2a).

**Table 2:** Most significant single-variant associations (*p* < 5 × 10^−7^) detected in the present GWAS.

SNP	REF allele	ALT allele	REF allele frequency^a,b^	*Z*-score^b^	*p*	Direction^b^	*I*^2^ (*p*)^c^	Position (chr:bp)	Position within gene	Gene symbol	Distance from gene (bp)
rs6035856	G	T	0.55	5.329	9.88 × 10^−8^	+++++++	0 (0.91)	20:2188542	intron	LOC388780	–
rs6035857	A	C	0.55	5.329	9.88 × 10^−8^	+++++++	0 (0.91)	20:2188544	intron	LOC388780	–
rs6082416	G	C	0.55	5.153	2.56 × 10^−7^	+++++++	0 (0.85)	20:2195832	downstream	LOC388780	2035
rs6137325	G	T	0.59	5.147	2.65 × 10^−7^	+++++++	0 (0.91)	20:2187943	exon	LOC388780	–
rs2094530	C	T	0.22	5.098	3.43 × 10^−7^	?--???-	0 (0.82)	13:51564457	downstream	GUCY1B2	4190
rs6047381	C	T	0.58	5.089	3.59 × 10^−7^	+++++++	0 (0.84)	20:2185357	upstream	LOC388780	2217
rs6137326	C	T	0.59	5.083	3.72 × 10^−7^	+++++++	0 (0.91)	20:2187944	exon	LOC388780	–
rs6132418	A	T	0.58	5.08	3.78 × 10^−7^	+++++++	0 (0.85)	20:2186281	upstream	LOC388780	1293

^a^Average allele frequency computed for reference (REF) alleles over all the datasets analyzed.

^b^Allele frequencies, meta-analysis *Z*-scores, and directions of effect refer to REF alleles. Directions of effect are reported for each single dataset analyzed, in the following order: AGS, Finland, France, Holland, Hungary, ENall1, ENall2. “+” means that the major allele is the risk allele, while “?” indicates that the variant was not tested in the corresponding dataset.

^c^*I*^2^ test for heterogeneity of genetic effect across datasets (the closer to “0”, the more homogenous is the genetic effect).

### Gene- and pathway-based enrichment analyses

Gene-level analysis of genome-wide single-variant association signals with DD revealed two significant enrichments, after correcting for the 18,013 genes tested across the genome (*p* < 2.8 × 10^−6^; Table S2c). These enrichments were observed for the gene *VEPH1* (*ventricular zone expressed PH domain-containing 1*; 3q25; *Z* = 5.63; permutation-based *p* = 8 × 10^−8^) and for the gene *LOC388780*, where top GWAS variant mapped to (*Z* = 5.26; *p* = 1.7 × 10^−7^). However, the analysis of 1329 canonical pathways from the MSigDB website did not reveal any significant enrichment (Table S2d).

### SNP-based heritability

We computed the SNP-based heritability (*h*^2^_SNP_) of DD through LD score regression, using the summary statistics of HapMap 3 SNPs analysed in the GWAS. This analysis yielded an estimate of *h*^2^_SNP_ (SE) = 0.19(0.06) on the observed scale, while, on the liability scale, we observed *h*^2^_SNP_ (SE) of 0.20(0.06) (assuming a dyslexia prevalence of 0.05) and of 0.25(0.08) (for prevalence 0.1; see Table S3a).

We next computed partitioned heritability for different functional categories in the genome, through stratified LD score regression. The analysis of 53 overlapping functional annotation categories in the baseline model (i.e., including functional annotations irrespective of the cell type) revealed no statistically significant enrichments of heritability for such general annotation classes (see Table S3b). Similarly, the stratified LD score regression applied to annotations specific to CNS cell types detected no significant contribution to SNP-based heritability of the four histone marks tested (H3K4me1, H3K4me3, H3K9ac, and H3K27ac; Table S3c). When we analysed partitioned heritability by sets of specifically overexpressed genes in 13 different brain regions available in the GTEx database (see “Methods” section), we observed no significant contributions to *h*^2^_SNP_ surviving correction for multiple testing (Table S3d).

### Polygenic scores and dyslexia risk

We report in Table 3 the results of the main PGS analysis on neuropsychiatric disorders, intelligence and educational attainment, including only variants with association *p* < 0.05 in the training GWAS (i.e. at *p*_*T*_ = 0.05), while the results at the different association significance (*p*_*T*_) thresholds tested are reported in Table S4a–h. At *p*_*T*_ = 0.05, glm logistic regressions revealed that standardized PGS of EDUyears and fluid intelligence were significantly associated with dyslexia risk in our sample, surviving correction for multiple testing, with OR = 0.86[0.82; 0.91] (*R*^2^ = 0.39%; *p* = 1.95 × 10^−7^) and 0.72[0.68; 0.76] (1.79%; 9.40 × 10^−29^), respectively. Also, we observed significant associations with dyslexia risk for three of the neuropsychiatric disorders analyzed: ADHD (1.23[1.16; 1.3]; 0.73%; 7.66 × 10^−13^), BD (1.53[1.44; 1.63]; 2.80%; 1.33 × 10^−43^) and SCZ (1.36[1.28; 1.45]; 1.35%; 3.65 × 10^−22^). Similarly, we identified a significant association with common genetic liability shared across different psychiatric disorders (1.23 [1.16; 1.30]; 0.69%; 3.12 × 10^−12^). These associations were concordant across all tested significance thresholds, and *p* values decreased when more inclusive criteria were used (i.e., for *p*_*T*_ ranging between 0.1 and 1, see Table S4a–h).

**Table 3:** Results of the polygenic score (PGS) analysis for the different training traits/disorders tested.

Trait/disorder PGS	OR [95% CI]	*R*^2^ (%)	*p*	Training GWAS (Reference)	Training GWAS *N* (cases/controls)
**ADHD**	**1.23 [1.16; 1.3]**	**0.73**	**7.66** **×** **10**^**−13**^	[67]	**53,293 (19,099/34,194)**
ASD	1.01 [0.96; 1.07]	<0.01	0.69	[68]	46,351 (18,382/27,969)
**BD**	**1.53 [1.44; 1.63]**	**2.80**	**1.33** **×** **10**^**−43**^	[66]	**51,710 (20,352/31,358)**
MDD	1.01 [0.95; 1.06]	<0.01	0.83	[70]	500,199 (170,756/329,443)
**SCZ**	**1.36 [1.28; 1.45]**	**1.35**	**3.65** **×** **10**^**−22**^	[69]	**77,096 (33,640/43,456)**
**Cross-Disorder**	**1.23 [1.16; 1.30]**	**0.69**	**3.12** × **10**^**−12**^	[61]	**438,997 (162,151/276,846)**
**EduYears**	**0.86 [0.82; 0.91]**	**0.39**	**1.95** × **10**^**−7**^	[63]	**766,345**
**Intelligence**	**0.72 [0.68; 0.76]**	**1.79**	**9.40** × **10**^**−29**^	[62]	**269,867**

We report odds ratios (OR) for dyslexia with 95% confidence intervals (95% CI) per standardized PGS in our dataset, along with relevant *R*^2^ and *p* values, at *p*_*T*_ = 0.05 in the training GWAS. Full results for the different *p*_*T*_ thresholds tested are reported in Table S4a–h. Statistically significant associations (*p* < 4.5 × 10^−4^) are highlighted in bold.

*ADHD* attention deficit hyperactivity disorder, *ASD* autism spectrum disorder, *MDD* major depressive disorder, *BD* bipolar disorder, *SCZ* schizophrenia, *CROSS-DISORDER* shared genetic basis of ADHD, ASD, BD, MDD, SCZ, anorexia nervosa, obsessive-compulsive disorder and Tourette syndrome based on the GWAS meta-analysis by the Cross-Disorder Group of the Psychiatric Genomics Consortium [61], *EduYears* years of education completed.

The analysis of PGS for SA and T of 34 brain cortical regions revealed an association of the transverse temporal gyrus T with prevalent DD risk, which remained significant after correction for multiple testing (OR = 0.90[0.86; 0.96]; *p* = 4.53 × 10^−4^; Table S4i). This association was confirmed in a multivariable setting, both in stepwise (0.90[0.85; 0.95]; *p* = 2.45 × 10^−4^; Table S4j) and in elastic net regression (OR = 0.92; Table S4k). However, the variance explained by this PGS was low (0.17% in univariate regression) and all the cortical PGS selected in elastic net regression jointly conferred only a modest gain in dyslexic classification accuracy, compared to the null model including only covariates (0.4%).

## Discussion

To the best of our knowledge, the present work reports the largest case-control GWAS study conducted on dyslexia to date, involving 2274 DD cases and 6272 controls from nine different populations of European ancestry, speaking six different languages.

We identified a suggestive association at rs6035856 (p~10^−8^), an intronic variant located within the gene *LOC388780* (20p13), ~400 bp downstream of exon 1. This small (~6 kb) gene encodes a non-coding RNA which has not been functionally characterized yet, but is expressed in different organs, including the CNS [52]. Gene-based association testing supported the implication of *LOC388780* in DD genetic risk, showing a genome-wide significant enrichment of associations for this gene. Based on the Roadmap Epigenome 25-state model using 12 imputed marks, this region is classified as a Promoter Upstream Transcription Start Site (*2_PromU* chromatin state) in several brain cell types, including those from middle hippocampus, anterior caudate, cingulate gyrus, inferior frontal lobe, and dorsolateral prefrontal cortex [78], suggesting potential roles in transcriptional regulation.

Gene-based analysis also detected significant evidence of enrichment for the gene *VEPH1* (*ventricular zone expressed PH domain-containing 1*; 3q25), coding for a partly characterized protein which promotes brain development [79], probably through regulation of the TGF-β signalling pathway [80]. However, we did not observe any significant enrichment of associations for TGF-β-related pathways.

The analysis of SNP-based heritability indicated that 20–25% of the total variance in DD could be explained by common variants in our dataset. This estimate is lower than typical heritability estimates for dyslexia provided by twin studies (40–60%) [2]. As with other complex traits, the discrepancy between twin- and SNP-based heritability suggests that part of dyslexia risk may be due to the genetic effects of variants other than SNPs, such as common copy number (CNVs) and rare variants. Although the relationship of CNVs and rare variants with DD and reading-related traits has not been extensively investigated to date, this hypothesis is partly supported by some recent findings. First, rare CNVs have often been implicated in familial forms of dyslexia [81] and the candidate gene *DYX1C1* was first identified through a rare chromosomal rearrangement which co-segregated with dyslexia in a Finnish family [82]. Similarly, a targeted high-throughput sequencing study of 96 reading-impaired subjects reported an excess of putatively damaging rare variants in the candidate susceptibility loci *DYX2* and *CCDC136/FLNC* [83]. Second, CNVs associated with neuropsychiatric disorders showed a significant influence on different cognitive traits in a large Icelandic population-based sample (*N*~102,000) [84]. In particular, a recurrent deletion of *15q11.2* was associated with a history of dyslexia and dyscalculia [84] and, in a later study, with cognitive, structural, and functional correlates of these impairments [85]. Third, a study reported >50% of the heritability of general cognition (IQ) and educational attainment (EduYears) to be explained by genetic variants in low LD with SNPs commonly genotyped on microarrays, especially rare variants. Indeed, SNP-based heritability of these traits approached the total heritability estimates from previous studies, when including also rare variants [86]. This suggests a substantial contribution of rare genetic variants to individual differences in intelligence and education, which may also extend to correlated cognitive traits such as reading ability.

PGS analyses revealed several significant associations between dyslexia risk and genetic liability to psychiatric disorders and other correlates.

First, we observed that PGSs for educational attainment and fluid intelligence were significantly associated with DD in our sample, in line with previous studies [30, 72, 87]. Luciano et al. [87] observed that PGSs of word reading, nonword repetition, and reading–spelling from GWAS studies of ~6600 children from UK and Australia showed significant positive associations with both verbal-numerical reasoning and educational attainment (college or university degree) in the UK Biobank cohort. Similarly, a PGS based on EduYears accounted for 2–5% of the variance in reading efficiency and comprehension in an independent UK sample (*N* = 5825) [72]. In the same study, Selzam et al. reported a PGSs of childhood general cognitive ability and adult verbal-numerical reasoning to explain a small but significant proportion (0.1–1.1%) of the variance in reading efficiency and comprehension at several developmental stages [72]. We later replicated these findings in a GWAS of reading-related cognitive skills partly overlapping with the present study (*N*_max_ = 3468), extending the evidence of genetic overlap to cognitive predictors of dyslexia risk like phoneme awareness and digit span [28]. More recently, a GWAS of word reading in 4430 US children presenting in hospitals/clinics provided a further replication, reporting higher fractions of variance explained by EduYears (18%) and intelligence PGSs (7%) [30]. Together, the various PGS-based studies strongly support the existence of shared genetic factors influencing educational attainment, general cognition, and more specialized abilities like reading [88].

Second, genetic liability to ADHD was significantly associated with an increased DD risk, explaining 0.73% of its variance (at *p*_*T*_ = 0.05). This finding is in line with the hypothesis of shared genetic bases between these disorders, initially suggested by twin studies [54, 56], and with evidence of genomic overlap reported for ADHD and the key cognitive features of dyslexia in our previous GWAS [28]. Recently, Price et al. [30] replicated the inverse association between ADHD-PGS and word reading in US children, as did Verhoef et al. [89] in a British longitudinal cohort (*N*_max_ = 5919) for reading accuracy/comprehension at age 7, reading and spelling accuracy at age 9.

Third, we detected genetic links between two other neuropsychiatric disorders—BD and SCZ—and dyslexia. Standardized BD- and SCZ-PGS were associated with an increased DD risk, explaining 2.8% and 1.4% of its variance, respectively. Comorbidity of DD with a number of psychiatric disorders—including also BD and SCZ—has been previously reported [59, 60], and siblings of dyslexic subjects showed a high relative risk of being affected by ADHD, BD, SCZ, depression and autism, among others [59]. Although no significant associations between a SCZ-PGS and continuous reading-related traits were observed in a smaller independent dataset [87], the association between SCZ genetic risk and DD is in line with the reported genetic influence of SCZ risk variants on reading problems in the general population [84]. To the best of our knowledge, no evidence of a common genetic basis for BD and reading difficulties has been reported so far, although shared familial (and potentially genetic) risks have been previously suggested [59, 90]. Of note, we detected no significant associations between MDD-/ASD-PGS and dyslexia, but we did observe this for psychiatric cross-disorder genetic liability. These findings open up new scenarios in psychiatric genetics, suggesting a shared genetic and biological foundation across many different neurodevelopmental and neuropsychiatric conditions of phenotypically and clinically different nature.

Finally, the analysis of PGS influencing different brain cortical regions revealed a small, but robust and significant, protective effect against DD risk for a PGS increasing thickness of the transverse temporal gyrus. This region, also known as Heschl’s gyrus, is located within the primary auditory cortex—which is fundamental for auditory discrimination and speech perception [91]—and has been previously implicated in dyslexia by neuroimaging evidence, although not always consistently across studies [92–95]. Moreover, it overlaps with the left perisylvian regions where Galaburda et al. detected neuronal ectopias in four post-mortem dyslexic brains [96]. Here, we provide evidence of a genetic overlap between dyslexia risk and potential brain structural features proposed from non-genetic studies, although caution is suggested in the interpretation of these findings due to the inconsistencies across neuroimaging studies and to the potential role of regional brain asymmetries in the measures analyzed, which here were not taken into account due to the unavailability of GWAS summary statistics for separate hemispheres [75].

In spite of strengths like the wealth of cohorts and languages analyzed, and a relative homogeneity of recruitment, phenotypic assessment, and QC procedures, the present study also shows some limitations. In particular, there was a non-optimal case:control ratio in some datasets and a lack of properly screened controls in the English-speaking datasets. Although we acknowledge these would be preferred to improve power, the use of unscreened population controls is common where large numbers are needed, and has been exploited elsewhere [38, 39, 97]. Indeed, while for very common diseases the use of unscreened controls may notably affect power, for less common disorders/statuses (with prevalence <0.2) the loss of power is reduced and counterbalanced by the larger sample size which can be achieved through the use of unscreened populations [98]. Also, the PGS approach is based on the assumption that population structure and other possible confounds are well controlled in the training and target GWAS, which we implemented by adjusting all analyses for sex and genetic ancestry. However, independent replication of these results is warranted to substantiate the novel findings coming from the PGS analysis. Moreover, although the present study represents to our knowledge the largest GWAS on dyslexia to date [98], its sample size is relatively low compared to other studies in the neuropsychiatric field [66–70], which limited the power of analyses. Larger collaborative efforts are being implemented to improve these aspects to further enlighten the genetic epidemiology of dyslexia.

## URLs

Wellcome Trust Case Control Consortium 2: https://www.wtccc.org.uk/ccc2/

PLINK: https://www.cog-genomics.org/plink2

QCTOOL: http://www.well.ox.ac.uk/~gav/qctool/

METAL: http://www.sph.umich.edu/csg/abecasis/Metal/index.html

MAGMA: http://ctg.cncr.nl/software/magma

MSigDB: http://software.broadinstitute.org/gsea/msigdb;

LD score regression: https://github.com/bulik/ldsc

Per-variant LD scores: https://data.broadinstitute.org/alkesgroup/LDSCORE/

Genotype-Tissue Expression portal (GTEx): http://www.gtexportal.org/home/

Brain eQTL Almanac (Braineac): http://www.braineac.org/

LocusZoom: http://www.locuszoom.org/

The R Project: https://www.r-project.org/

MASS package: https://cran.r-project.org/web/packages/MASS/index.html

Glmnet package: https://cran.r-project.org/web/packages/glmnet/index.html

Caret package: https://cran.r-project.org/web/packages/caret/index.html

## Supplementary information

Supplementary File S1

Supplementary File S2

Supplementary File S3

Supplementary File S4

## Data Availability

Summary statistics data supporting the findings of the present study are available upon request to the corresponding authors.

## Code availability

Bioinformatic codes supporting the findings of the present study are available upon request to the corresponding authors.
