# Supplementary material for: Genome-wide association study reveals new insights into the heritability and genetic correlates of developmental dyslexia
Source: Mol Psychiatry. 2020 Oct 14;26(7):3004–17. doi: 10.1038/s41380-020-00898-x (PMC8505236; doi:10.1038/s41380-020-00898-x)
Supplement: Supplementary file 1 — Supplementary File S1 [file 41380_2020_898_MOESM1_ESM.docx]

**Supplementary Methods**

***Definition of dyslexia phenotype***

DD cases were generally defined as participants scoring ≤-1.25 standard deviations (SDs) in a standardized word reading test (see below), while controls were generally defined as individuals with word reading scores >-0.85 SDs [1, 2]. An exception was represented by controls of the English-speaking datasets, which were drawn from the British general population (Wellcome Trust Case Control Consortium 2) [3] and by 148 German cases, which were instead defined using an age-appropriate spelling test (writing to dictation) [4, 5]. For these children, an expected spelling score was calculated on the basis of an assumed correlation of *r*=0.4 between the proband's IQ and spelling ability [4, 5]. Then, children were classified as ‘affected’ when the observed spelling score and the one expected based on IQ showed a discrepancy of ≥1.5 SDs in the initial sample.

In Austria, Germany, Switzerland, Finland, France, Hungary and the Netherlands, reading accuracy and speed for words were assessed by presenting language-specific material under a speeded instruction (“Read as quickly as possible without making mistakes”). The number of words read correctly per minute was then converted into Z-scores based on grade appropriate norms, within each country (see [2, 6] for details). In the British cases, an untimed word reading task was administered, which was grade-normed and standardized as above. In the Colorado cases, a composite (average) measure of timed and untimed word reading was used, which was age-adjusted and standardized against the normative mean of a matching control population (see [7] for details).

**Table S1a.** Main genotype QC statistics and information for each dataset involved in the study.

| Dataset | N  (before/after QC) | Sex Ratio  M:F  (unknown) | Genotyping platform | SNPs | SNPs After QC | SNPs after  imputation |
| --- | --- | --- | --- | --- | --- | --- |
| AGS | 1,502/1,454 | 886:568 | Illumina HumanHap 300k | 317k ^a^ | 292,056 ^a^ | 5,747,699 |
|  |  |  | Human CoreExome | 543k | 240,130 |  |
| Finland | 336/324 | 167:157 | Human CoreExome | 543k | 243,282 | 6,197,696 |
| France | 165/163 | 94:69 | Illumina HumanHap 660k | 660k | 471,468 | 6,177,173 |
| Hungary | 243/241 | 136:105 | Human CoreExome | 543k | 236,643 | 5,948,732 |
| Netherlands | 311/284 | 157:127 | Human CoreExome | 543k | 236,871 | 5,978,155 |
| ENall1 ^c^ | 3,531/3,313 | 1,773:1,540 | Illumina Human OmniExpress | 683k ^b^ | 275,819 | 6,005,089 |
|  |  |  | Illumina HumanHap 550k | 550k |  |  |
|  |  |  | Illumina 1.2M | 1,158k |  |  |
| ENall2 ^c^ | 2,947/2,767 | 1426:1337  (4) | Illumina HumanHap 300k | 317k | 291,161 | 6,058,845 |
|  |  |  | Illumina HumanHap 550k | 550k |  |  |
|  |  |  | Illumina 1.2M | 1,158k |  |  |

^a^ A subset of the German sample (N=195) was genotyped on the Illumina 317k chip and shared a low number of SNPs with the Human CoreExome array. These samples were thus QCed separately and merged with the rest of the AGS samples only after imputation.

^b^ SNPs in these datasets were preliminarly filtered through Illumina GenomeStudio software before producing hard-call genotype data, as described by [7].

^c^ Since the English-speaking cases had been genotyped on four different platforms and the number of SNPs in common was too low to allow an imputation of good quality (<160k SNPs), we decided to split these samples into two case-control datasets merging them with two distinct WTCC2 control cohorts, namely WTCC2_1958 and WTCC2_NBS [3] (see Table 1 in the manuscript for details). Only variants which were shared among the multiple arrays used within each dataset were retained for the following genotype QC and imputation.

**Table S1b.** Details on genotype QC and imputation of the datasets involved in the study.

| Filter | Sibling-based datasets | All other datasets |
| --- | --- | --- |
| Individual genotyping rate | > 98 % | |
| Genetic relatedness (PI-HAT) | among unrelated subjects: PI-HAT < 0.2;  among related subjects: PI-HAT in range [0.2 ; 0.65[ and concordant with pedigree based information ^a^ | < 0.0625 |
| Duplicates (PI-HAT) | ≤ 0.65 | |
| Sex mismatches with pedigree-based info | based on X chromosome genetic data  (PLINK v1.9 default settings) | |
| Genetic ancestry: distance in first two MDS components from mean ^b, c^ | < 4 SD | < 5 SD |
| Genome-wide heterozygosity:  deviation of autosomal heterozygosity from mean ^c^ | < 3 SD | < 4 SD |
| Minor allele frequency ^c^ | ≥ 5 % | |
| Variant call rate ^c^ | ≥ 98 % | |
| HWE test *p*-value ^c, d^ | ≥ 10^-6^ | |
| Variants on non-autosomal chromosomes | removed | |
| Ambivalent SNPs (A/T and G/C) ^e^ | removed | |
| Variants not present in the 1000 genomes phase I v3 EUR reference panel | removed | |
| IMPUTE2 info metric | ≥ 0.8 | |

Sibling-based datasets: related cases from UK and US + unrelated controls from UK (ENall1); all other datasets: Austria-Germany-Switzerland (AGS), Finland, France, Hungary, the Netherlands and unrelated cases and pseudo-controls from UK (ENall2).

^a^ In ENall1, samples showing PI_HAT values discordant with pedigree-based information were filtered out (i.e. unrelated subjects showing PI-HAT ~0.25-0.5; related subjects showing PI-HAT <0.2; and subjects showing PI-HAT ~1).

^b^ MDS components of the genetic distance matrix were calculated through PLINK 1.9. First, we used the QCed and imputed genetic data to build an IBS/IBD similarity matrix for all the subjects within each dataset (through the –genome command); then we extracted the first 10 MDS components from the IBS/IBD matrix (through the –cluster –mds-plot commands).

^c^ These filters were applied both before and after imputation.

^d^ Hardy Weinberg Equilibrium (HWE) test was applied to cases and controls jointly, under the assumption of polygenic inheritance of dyslexia susceptibility with no strong monogenic effects by the common variants tested in the study. Therefore, it should be considered as a filter for technical genotyping and calling errors.

^e^ Note on allele strands: all genotype data were aligned to the plus/minus strand orientation before imputation, while ambiguous SNPs (A/T and G/C) were removed before imputation, and only non-ambiguous alleles present in the 1000 Genomes reference panel were kept, which is also in plus/minus orientation. This ensures that all imputed data sets were unambiguously harmonized to the plus/minus alignment for the meta-analysis.

***Power and sample size estimation analysis***

We performed a power and sample size estimation analysis using the Genetic Power Calculator [8]. Specifically, we computed the power (1-β) to detect genome-wide significant associations with a binary trait (α=5×10^-8^), in a case-control setting involving 2,274 unrelated cases and a case:control ratio of 2.76, corresponding to our study. We assumed a genetic additive model and perfect linkage disequilibrium for potential tagging variants of the causative SNPs, and two alternative prevalence values of the disease analysed, namely 5% and 10%, representing the extreme values of the prevalence range commonly reported in the literature [9]. For each of these conditions, we computed power for different MAFs (0.05, 0.1, 0.2, 0.5) and increases in relative risk associated with each copy of the effect allele (10%, 20%, 30%, 40%, 50%). For each simulated scenario, the number of cases necessary to reach an 80% power was computed, assuming a constant case:control ratio, as in our analysis (2.76). The results of this analysis are reported in Table S1c, d below.

**Table S1.** Results of power and sample size estimation analysis for the study, assuming dyslexia prevalence of **c)** 5% and **d)** 10%.

c)

| Power  (# cases) | Increase of risk per effect allele copy | | | | |
| --- | --- | --- | --- | --- | --- |
| MAF | 10% | 20% | 30% | 40% | 50% |
| 0.05 | <0.01 (57,936) | 0.09 (15,056) | 2.62 (6,948) | 21.4 (4,054) | 62.46 (2,689) |
| 0.1 | 0.01 (31,109) | 1.27 (8,217) | 25.27 (3,851) | 79.71 (2,281) | 98.81 (1,534) |
| 0.2 | 0.04 (18,105) | 10.6 (4,935) | 75.54 (2,382) | 99.36 (1,450) | 100 (1,000) |
| 0.5 | 0.18 (12,793) | 26.31 (3,802) | 90.45 (1,980) | 99.86 (1,290) | 100 (946) |

d)

| Power  (# cases) | Increase of risk per effect allele copy | | | | |
| --- | --- | --- | --- | --- | --- |
| MAF | 10% | 20% | 30% | 40% | 50% |
| 0.05 | <0.01 (51,805) | 0.15 (13,416) | 4.34 (6,171) | 31.3 (3,590) | 75.87 (2,374) |
| 0.1 | 0.01 (27,830) | 2.08 (7,329) | 35.75 (3,426) | 89.1 (2,024) | 99.71 (1,358) |
| 0.2 | 0.07 (16,211) | 16.03 (4,410) | 85.66 (2,125) | 99.85 (1,292) | 100 (890) |
| 0.5 | 0.29 (11,484) | 36.1 (3,414) | 95.47 (1,779) | 99.97 (1,159) | 100 (851) |

Power (%) of each scenario simulated is reported, along with the number of cases required to reach an 80% power for the analysis, under a constant case:control ratio of 2.76. Abbreviations: MAF = Minor Allele Frequency.

**Figure S1.** Quantile-quantile (QQ) plot of the GWAS meta-analysis.

**
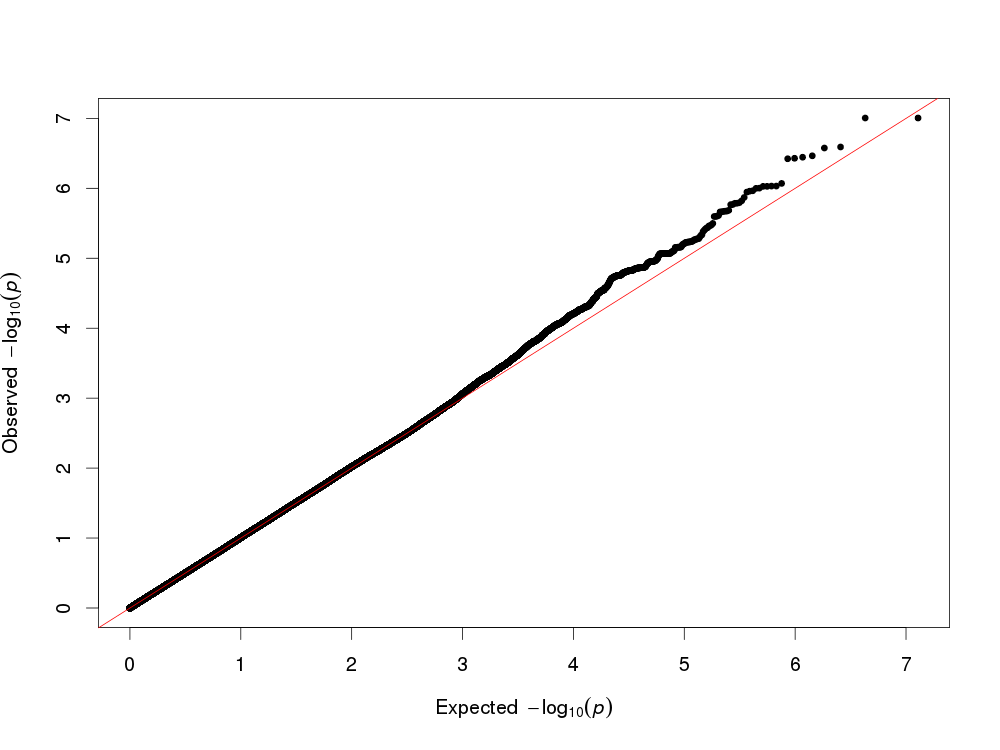
**

**References**

1. Landerl K, Ramus F, Moll K, Lyytinen H, Leppänen PHT, Lohvansuu K, et al. Predictors of developmental dyslexia in European orthographies with varying complexity. J Child Psychol Psychiatry. 2013;54:686–694.

2. Moll K, Ramus F, Bartling J, Bruder J, Kunze S, Neuhoff N, et al. Cognitive mechanisms underlying reading and spelling development in five European orthographies. Learn Instr. 2014;29:65–77.

3. Rucker JJH, Breen G, Pinto D, Pedroso I, Lewis CM, Cohen-Woods S, et al. Genome-wide association analysis of copy number variation in recurrent depressive disorder. Mol Psychiatry. 2013;18:183–189.

4. Schulte-Körne G, Ziegler A, Deimel W, Schumacher J, Plume E, Bachmann C, et al. Interrelationship and familiality of dyslexia related quantitative measures. Ann Hum Genet. 2007;71:160–175.

5. Roeske D, Ludwig KU, Neuhoff N, Becker J, Bartling J, Bruder J, et al. First genome-wide association scan on neurophysiological endophenotypes points to trans-regulation effects on SLC2A3 in dyslexic children. Mol Psychiatry. 2009;16:97.

6. Landerl K, Ramus F, Moll K, Lyytinen H, Leppanen PH, Lohvansuu K, et al. Predictors of developmental dyslexia in European orthographies with varying complexity. J Child Psychol Psychiatry. 2013;54:686–694.

7. Gialluisi A, Newbury DF, Wilcutt EG, Olson RK, DeFries JC, Brandler WM, et al. Genome-wide screening for DNA variants associated with reading and language traits. Genes Brain Behav. 2014;13:686–701.

8. Purcell S, Cherny SS, Sham PC. Genetic Power Calculator: design of linkage and association genetic mapping studies of complex traits. Bioinformatics. 2003;19:149–150.

9. Pennington BF, Bishop DVM. Relations Among Speech, Language, and Reading Disorders. Annu Rev Psychol. 2009;60:283–306.
